# Marijuana Poisoning in Canines in the Aburrá Valley (Antioquia-Colombia), 2023-2024

**DOI:** 10.1155/vmi/4844163

**Published:** 2025-01-06

**Authors:** Natalia Restrepo Marulanda, José Fernando Ortiz Álvarez, Jaime Humberto Londoño Puerta, Angélica María Blandón Peralta, Natalia Uribe Corrales

**Affiliations:** Group of Veterinary Investigation GIVET, Program of Veterinary Medicine, Lasallian University Corporation, Bogotá, Colombia

**Keywords:** cannabis, dogs, intoxication, tetrahydrocannabinol

## Abstract

**Background and Aim:** Marijuana poisoning in canines is still considered a taboo topic. Poisoning in pets is becoming increasingly common, in many cases resulting in severe clinical signs, such as ataxia, urinary incontinence, mydriasis, depression, and hyperesthesia. Colombia does not have any reliable test for the diagnosis of exposure to cannabis in animals, and it is not an obligation to report this kind of poisoning to the authorities. Therefore, the main objective of this study was to determine the frequency of cases compatible with tetrahydrocannabinoid (THC) poisoning in canines in the Aburrá valley (Antioquia-Colombia), 2023-2024.

**Materials and Methods:** A cross-sectional study was conducted in the Aburrá valley (Antioquia-Colombia) from 2023 to 2024. Veterinary centers that were allowed to participate were visited, and the clinical records were examined on the canines that were diagnosed positive for marijuana poisoning. We examined the clinical record for different risk factors that could explain the poisoning. A descriptive statistic and a Chi-square test were used to identify risk factors; *p* < 0.05 were considered statistically significant.

**Results:** One hundred and thirteen (113) cases compatible with cannabis poisoning were found in dogs. Puppies, crossbreeds, trim sizes, and canines whose owners have middle or high economic incomes were the most affected. In addition, a relation between the route of possible cannabis poisoning and the severe presentation of clinical signs was found, with ingestion and inhalation being the routes that generated a more pronounced sign. Likewise, a relationship between age and the most probable route of exposure was found, finding that puppies were more related to the ingestion of cannabis products.

**Conclusion:** This is the first article in Colombia that was focused on determining the frequency of possible cannabis poisoning in canines. The dogs that are more susceptible to suffering cannabis poisoning are puppies and small-size dogs that are attended by veterinary medical centers and are in neighborhoods that are recognizable by people who have high salaries.

## 1. Introduction

Marijuana is a plant that, throughout the history of humanity, has been used for both medicinal and recreational purposes [1]. Tetrahydrocannabinol (THC) is the main component responsible for the psychoactive effects [2]. In recent years, marijuana has become the object of considerable public health and policy discussions due to the changes legalizing this component in many countries, including Colombia [3].

Cannabis use is becoming increasingly common, whether recreational or medicinal, among humans, representing a greater susceptibility toward canines to present an accidental exposure. The illegal distribution and commercialization of edible products, such as cookies, brownies, gummies, beer, and other products, make them more attractive for consumption, which predicts an increase in incidents of poisoning of domestic animals, particularly dogs [4].

Concerning companion animals, canines are the most exposed to marijuana through the ingestion of the plant or items made with it. Many of the poisonings are not lethal; however, they result in severe clinical signs, such as ataxia, urinary incontinence, mydriasis, depression, and hyperesthesia, which means that veterinarians must treat them [3, 5].

Some dog characteristics related to accidental poisoning are breed, sex, size, and reproductive status [6]. Unfortunately, in the Colombian veterinary medical environment, there is no obligation to report events of this kind of poisoning. In addition, in Colombia, we do not have any reliable test for the diagnosis of exposure to cannabis in animals [6]. Therefore, the objectives of this study were to establish the frequency of cases compatible with tetrahydrocannabinoid (THC) poisoning in canines in the Aburrá valley (Antioquia-Colombia) in 2023-2024.

## 2. Materials and Methods

### 2.1. Ethical Approval

The Ethics Committee for Experimentation with Animals of the Lasallian University Corporation approved this research in February 2023. All clinics that the authors visited were informed about the process we were doing; in addition, we never used personal information from the records because the personal information was not available to us (clinics did not share the name of the owner, telephone or cellphone number, or addresses) to protect the privacy of the owner and pet. All the records were working in an Excel spreadsheet, and we used a code to let anyone know which clinic was participating in this study.

### 2.2. Period and Area of Study

The study was conducted from March 2023 to July 2024.

**Figure 1:**
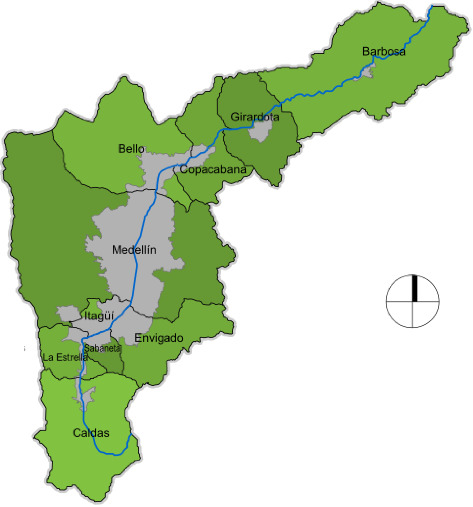
Map of the Aburrá valley, Antioquia, Colombia [7].

The Aburrá valley is a geographic region located in the south-central department of Antioquia (Colombia), at the heart of the Central Andes Mountains. It includes 10 municipalities of Antioquia (Barbosa, Girardota, Copacabana, Bello, Medellín, Envigado, Itagüí, Sabaneta, La Estrella, and Caldas) (Figure 1).

### 2.3. Study Design

A cross-sectional study was carried out, reviewing medical records of veterinary centers that agreed to participate in the project and whose canine patients had THC poisoning as a presumptive diagnosis between 2023 and 2024.

### 2.4. Sampling

No sampling was carried out; instead, work was performed with all the veterinary centers that wanted to participate. It was verified that concerting veterinary centers signed a participation acceptance form autonomously, explaining the project, the information used, and the risks and benefits. After this, information was only taken from medical records, with more than 80% completed correctly. The medical record had to report that the canine had at least two signs and that the canine responded to the treatment to consider that the dog effectively could be poisoned by THC.

### 2.5. Statistical Analysis

Analysis was performed using Stata software Version 15 (StataCorp et al., USA).

The relative frequencies summarized the demographic characteristics and clinical signs of the dogs with a diagnosis compatible with TCH poisoning.

Pearson's Chi-square test detected an association between demographic characteristics, clinical signs, and poisonings consistent with TCH.

Finally, clusters were generated according to the municipality where the veterinary center is located to identify risk groups for possible poisoning with cannabinoid products.

## 3. Results

### 3.1. Demographics

**Figure 2:**
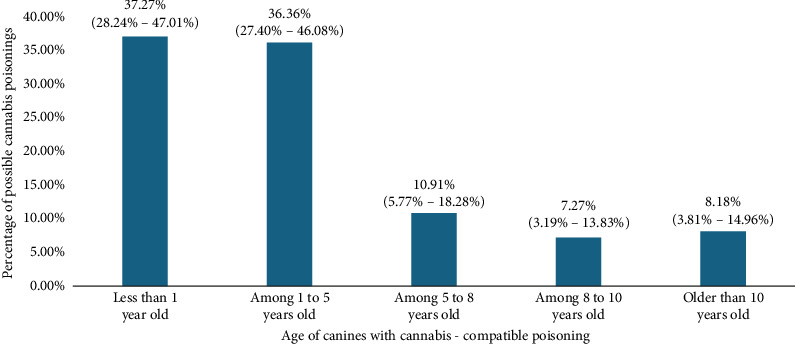
Age of canines with cannabis-compatible poisoning.

After the review of 349, it was found that a total of one hundred thirteen (113) cases were compatible with cannabis poisoning, representing a general frequency of compatible poisoning of 32.37% (CI: 25.69%–43.61%). No differences were detected among the number of poisoning cases reported during the years which were explored. In addition, it was evidenced that poisoning was higher in puppies (less than 1 year) (see Figure 2) and similar among sexes (male vs. female); small sizes were also the most affected (see Table 1).

**Figure 3:**
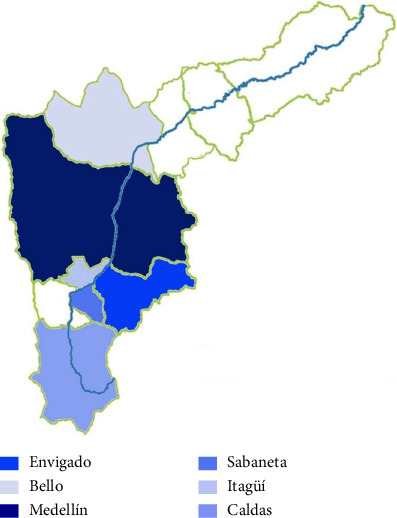
Most affected areas of the Aburrá valley.

Moreover, cannabis consumption in this study reflected that the most affected social classes are the middle and upper classes, being the most affected municipalities (Medellín, El Poblado locality with 38.05%, and Envigado, La Buena Mesa locality with 17.70%), see Figure 3.

### 3.2. Routes of Exposure and Diagnosis

**Figure 4:**
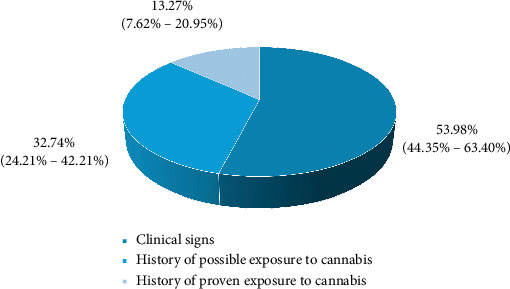
Diagnostic methods in poisoning compatible with cannabis.

**Figure 5:**
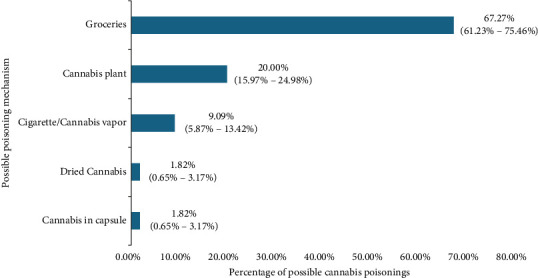
Possible poisoning mechanism in canines in Aburrá valley.

As shown in Figures 4 and 5, the primary mechanism for diagnosing cannabis-compatible poisoning was clinical signs. However, among those dogs that had possible poisoning due to a history of proven exposure to cannabis, it was known that 67.27% (CI: 61.25%–73.48%) were due to having eaten groceries that contained cannabis.

### 3.3. Clinical Signs and Treatments

**Figure 6:**
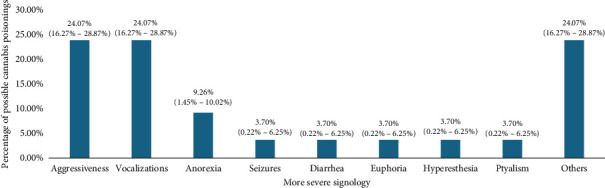
More severe signology in canines in Aburrá valley.

Within the signs manifested by canines, the most common were lethargy, ataxia, disorientation, vomiting, and mydriasis, as seen in Table 2. However, when investigating which signs the canines presented with greater severity, it was reported that they were mainly aggressiveness and vocalizations, as shown in Figure 6. Likewise, it was found that 50.44% (CI: 46.53%–54.65%) of the patients were hospitalized between 6 and 24 h to be treated by veterinary medical professionals.

It was found that there was an association between the route of possible cannabis poisoning and the severe presentation of clinical signs, with ingestion and inhalation being the routes that generated a more pronounced signology (*x*^2^: 8.04 and *p*=0.049). Likewise, a relationship was found between age and the most probable route of exposure, finding that those under 1 year of age were more related to the ingestion of cannabis products. In comparison, those over 1 year of age were more related to the inhalation of cannabis products (*x*^2^: 14.20 and *p*=0.002).

## 4. Discussion

Currently, the situation regarding cannabis consumption in Colombia is regulated using the minimum dose, which indicates that “a dose for personal use is the amount of marijuana that does not exceed twenty (20) grams and that over marijuana hashish which does not exceed five (5) grams” [8]. Furthermore, “Law 30 of 1986, regulated in its article 32, the criminalization of the cultivation, conservation or financing of marijuana plantations (number greater than twenty (20) plants)” [9]; therefore, based on the information above, the cultivation of this plant is allowed, but people cannot exceed 20 gr.

In addition, Colombia allows the marketing of cannabis-based products, whether edible (such as gummies, liqueurs, brownies, and cookies), cosmetics, supplements, creams, ointments, and other products. This law establishes the regulatory framework for producing, distributing, marketing, exporting, and importing cannabis and its derivatives for medicinal and scientific purposes [10]. Due to the element mentioned, with current trends in cannabinoid legalization and reduced or eliminated state penalties for cannabis possession, the odds of cannabis poisoning have increased [3, 11].

The study showed that the most common route of exposure for this poisoning was through edibles, with a percentage of 67.27%, comparing it with consumption of the cannabis plant at 20.00%, cigarette inhalation/cannabis vapor at 9.09%, dry cannabis consumption at 1.82%, and capsule cannabis consumption at 1.82%; this result is possibly related because people have developed different forms of oral presentation for cannabis, mixing it with various foods, which makes them more attractive to consumers: canines, especially young ones [12].

In contrast to other studies, this article found that neutered dogs had fewer reports of poisoning compatible with cannabis than intact canines [12–14]; this could be explained because the most common age range was less than 1 year. Therefore, sterilization is frequently made in canines older than 1 year since some studies have suggested that neutering before the first year of life may be associated with long-term health risks in dogs. Some experts recommend waiting until animals have reached physical maturity [15, 16].

Similarly, the canines with the highest probability of possible cannabis poisoning were young and small dogs [14, 17–20], possibly because these canines, being smaller, have less body surface area and such a low age range and their metabolism is not fully developed. Therefore, their lethal dose is lower, making them more susceptible than other breeds with a larger body surface area and an older age range. It may also be because these animals can live more closely and intimately with their owners, which leads to greater exposure to drugs in their environment [19].

Regarding the socioeconomic finding, this research has found results that are different from other authors in which cannabis poisoning is more related to the low-income class [21, 22]. In this study, the result was that it was bounteously related to high income, which may be related to access to economic resources that facilitate the purchase, because of the cost on cannabis, can be significant [23]. In addition, this difference can be explained by cannabis products availability in urban markets; nonetheless, this link to its point that should be further explored in future research [24]. Moreover, social networks and social pressure are also critical regarding this issue because cannabis consumption predominates more in a population between 18 and 25 years old, affecting young population with higher education mainly [25].

Finally, it is important to improve veterinary practice that laboratories, universities and governments conduct more efforts in research to improve the diagnosis of this kind of poisoning due to the hard effort and the less of a gold standard mechanism to identify TCH poisoning in animals due to actual tests properly can give us many false negative results [26]. At the moment, it is important that the veterinary staff be able to correctly identify possible poisoning, analyze the clinical signs, and make a deeper anamnesis to identify elements that could help us suspect contact from the canine to TCH.

## 5. Conclusion

To the best of the author's knowledge, this is the first article in Colombia that was focused on determining the frequency of possible cannabis poisoning in canines. It provides information on the characteristics (demographics, clinical signs, and diagnostic methods) that could help construct different laws related to drug consumption.

The dogs that are more susceptible to suffering cannabis poisoning are puppies and small-size dogs that are attended by veterinary medical centers that are located in neighborhoods that are recognizable by people who have high-salary lives.

Making a deep anamnesis is crucial in poisoning cases; the veterinary medical team must give education to owners about the importance of not omission the circumstances and history of the pet because it could cause a wrong diagnosis; and the primary way to diagnose cannabis poisoning is through the history reported by owners.

### 5.1. Limitations of the Study

When considering these results, there are elements to be considered, such as the following:

• Relying solely on data from veterinary centers that participated voluntarily could be a selection bias. Therefore, our findings may not fully represent the situation across the region, particularly in rural or low-income areas that were not included.
• The main challenge of this study was the absence of reliable diagnostic tests to confirm cannabis exposure in animals in Colombia. The diagnosis was based on clinical signs; this could introduce the possibility of misidentification of cases.
• In theory, the most valuable information to predict the probability of cannabis poisoning in dogs is to be able to verify the presence of this element in the canine's environment; however, these data are not available.

## Figures and Tables

**Table 1:** Characterization of canines with possible cannabis poisoning.

	Characteristic	Frequency	Percent (%)	Exact 95% LCL (%)	Exact 95% UCL (%)
Gender	Male	61	54.95	45.22	64.41
	Female	50	45.05	35.59	54.78

Neutered	Yes	29	29.59	20.79	39.66
	No	69	70.41	60.34	79.21

Breed	Crossbreed	32	22.12	14.86	30.90
	French bulldog	18	13.27	7.62	20.95
	Beagle	10	8.85	4.33	15.67
	Husky	10	8.85	4.33	15.67
	Pomeranian	10	8.85	4.33	15.67
	Cocker spaniel	7	6.19	2.53	12.35
	Pitbull	5	4.42	1.45	10.02
	Pincher	4	3.54	0.97	8.82
	Poodle	3	2.65	0.55	7.56
	Labrador retriever	2	1.77	0.22	6.25
	German shepherd	2	1.77	0.22	6.25
	Australian shepherd	2	1.77	0.22	6.25
	Teckell	2	1.77	0.22	6.25
	Others^∗^	6	0.88	2.53	12.35

^∗^The breeds in this category correspond to: Boston terrier, shepherd collie, Pomsky, Colombian hound, Shih-Tzu, and Yorkshire terrier.

**Table 2:** The most common clinical signs of cannabis intoxication.

Signs	Frequency	Percent (%)	Exact 95% LCL (%)	Exact 95% UCL (%)
Lethargy	66	58.41	48.76	67.60
Ataxia	60	53.10	43.48	62.55
Disorientation	59	52.21	42.61	61.70
Vomit	53	46.90	37.45	56.52
Mydriasis	50	44.25	34.91	53.89
Hypothermia	45	39.82	30.73	49.46
Tremors	39	34.51	25.82	44.04
Anorexia	36	31.86	23.41	41.29
Anxiety	36	31.86	23.41	41.29
Diarrhea	35	30.97	22.61	40.36
Bradycardia	34	30.09	21.82	39.43
Agitation	31	27.43	19.46	36.63
Polydipsia	30	26.55	18.68	35.68
Vocalizations	30	26.55	18.68	35.68
Urinary incontinence	29	25.66	17.91	34.74
Seizures	27	23.89	16.37	32.83
Hyperesthesia	27	23.89	16.37	32.83
Ptyalism	27	23.89	16.37	32.83
Respiratory depression	26	23.01	15.61	31.87

## Data Availability

The data that support the findings of this study are available from the corresponding author upon reasonable request.
